# Isolation and characterization of an exopolygalacturonase from *Fusarium oxysporum *f.sp. *cubense *race 1 and race 4

**DOI:** 10.1186/1471-2091-12-51

**Published:** 2011-09-15

**Authors:** Zhangyong Dong, Zhenzhong Wang

**Affiliations:** 1 Laboratory of Physiological Plant Pathology, South China Agricultural University, Guangzhou 510642, People's Republic of China; 2 Zhongkai University of Agriculture and Engineering, Guangzhou 510225, People's Republic of China

## Abstract

**Background:**

Fusarium wilt is an economically devastating disease that affects banana production. Although Cavendish banana cultivars are resistant to *Fusarium oxysporum *f.sp. *cubense *race 1 (FOC1) and maitain banana production after Gros Michel was destructed by race 1, a new race race 4 (FOC4) was found to infect Cavendish.

**Results:**

An exopolygalacturonase (PGC2) was isolated and purified from the supernatant of the plant pathogen *Fusarium oxysporum *f.sp. *cubense *race 4 (FOC4). PGC2 had an apparent Mr of 63 kDa by SDS-PAGE and 51.7 kDa by mass spectrometry. The enzyme was *N*-glycosylated. PGC2 hydrolyzed polygalacturonic acid in an exo-manner, as demonstrated by analysis of degradation products. To obtain adequate amounts of protein for functional studies between the PGC2 proteins of two races of the pathogen, *pgc2 *genes encoding PGC2 from race 4 (FOC4) and race 1 (FOC1), both 1395 bp in length and encoding 465 amino acids with a predicted amino-terminal signal sequence of 18 residues, were cloned into the expression vector pPICZaA and then expressed in *Pichia pastoris *strains of SMD1168. The recombinant PGC2 products, r-FOC1-PGC2 and r-FOC4-PGC2, were expressed and purified as active extracellular proteins. Optimal PGC2 activity was observed at 50°C and pH 5. The *K*_m _and *V*_max _values of purified r-FOC1-PGC2 were 0.43 mg.mL^-1 ^and 94.34 units mg protein^-1 ^min^-1^, respectively. The *K*_m _and *V*_max _values of purified r-FOC4-PGC2 were 0.48 mg.mL^-1 ^and 95.24 units mg protein^-1 ^min^-1^, respectively. Both recombinant PGC2 proteins could induce tissue maceration and necrosis in banana plants.

**Conclusions:**

Collectively, these results suggest that PGC2 is the first exoPG reported from the pathogen FOC, and we have shown that fully functional PGC2 can be produced in the *P. pastoris *expression system.

## Background

The banana (*Musa *spp.) is one of the world's most popular fruits and is regarded as the fourth most important crop in developing countries [1]. It suffers from several diseases, the most famous being Fusarium wilt disease (Panama disease), which is regarded as one of the most significant threats to banana production worldwide [2]. This disease is caused by the fungus *Fusarium oxysporum *f. sp. *cubense *(FOC) and has been reported in all banana-growing regions of the world, including Australia, Asia, Africa and Central and South America [3].

FOC has been classified into four physiological races based on pathogenicity to host cultivars in the field. FOC1 infects the cultivar Gros Michel; FOC2, 'Bluggoe'; FOC3, *Heliconia *spp.; and FOC4, Cavendish cultivars and all cultivars susceptible to FOC1 and FOC2 [4]. Earlier in the last century, FOC1 infection nearly destroyed the world's banana industry, which was based on the Gros Michel cultivar. Consequently, Gros Michel was replaced by Cavendish cultivars, which were resistant to FOC1. However, FOC4, which is capable of attacking Cavendish cultivars, was reported in Taiwan and Africa in 1967. To date, FOC4 has caused serious crop losses in Asia, Australia and Africa [5]. Grimm (2008) predicted that if FOC4 hits the banana heartland in Latin America, it could be game over for banana production in the region [6].

The plant cell wall is a barrier to the penetration and spread of phytopathogenic bacteria and fungi, so many plant pathogens produce extracellular enzymes that can degrade cell wall polymers. Cell wall-degrading enzymes and their genes have been studied for their possible role in many aspects of pathogenicity, including penetration, maceration, nutrient acquisition, plant defense induction, and symptom expression [7]. Polygalacturonases (PGs) are pectic enzymes that hydrolyze polygalacturonan, and they are the key components of pectinases. PGs are further classified into endoPGs and exoPGs, although some enzymes exhibit both endo- and exoPG activities [8]. EndoPGs (EC 3.2.1.15) cleave the backbone of polygalacturonan internally, whereas exoPGs (EC 3.2.1.67) hydrolyze monomers progressively from the nonreducing end of the substrate. ExoPGs have been reported in plant-pathogenic fungi [9] and their role in disease has been studied in the fungal plant pathogens *Cochliobolus carbonum *[10] and *Fusarium oxysporum *f.sp. *lycopersici *[11]. ExoPGs may have an important function in pathogen-plant interactions because they degrade oligogalacturonides released by endoPGs to elicitor-inactive monomers [12]. Moreover, exoPGs are not subject to inhibition by plant polygalacturonase-inhibiting proteins (PGIPs) [13].

In this study, we report for the first time the isolation and purification of an exoPG (PGC2) from the supernatant of the plant pathogen FOC4. We cloned the *pgc2 *genes of FOC1 and FOC4 and then expressed them in *P. pastoris*. Both recombinant PGC2 proteins retained their exoPG activity. Further studies of these genes will provide valuable insights into the role of PGC2 in FOC pathogenicity in banana cultivars.

## Results and Discussion

### Purification of PGC2

**Figure 1:**
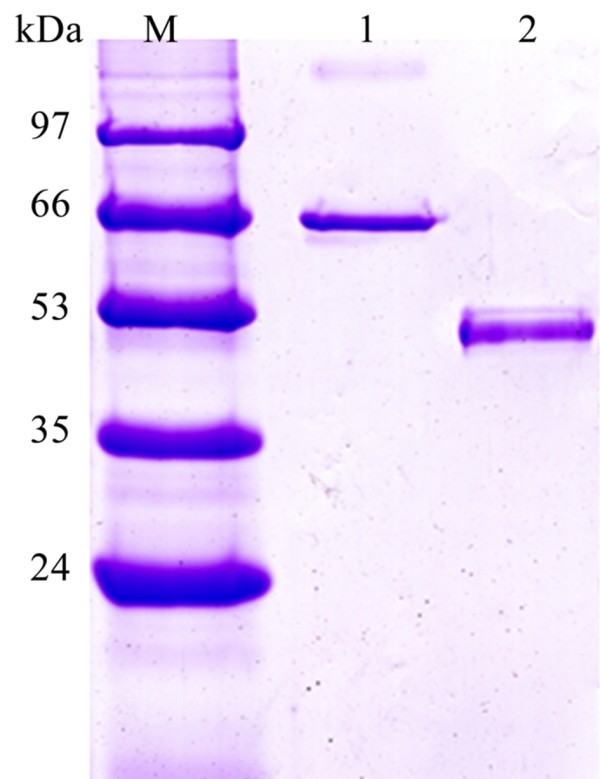
**SDS-PAGE analysis of purified PGC2 from *F. oxysporum *f. sp. *cubense *race 4 (FOC4)**. Lane M: protein marker. Lane 1: purified PGC2. Lane 2: PGC2 after treatment with N-glycosidase F.

PG activity in the FOC4 culture supernatant could be detected when the fungi were grown in the presence of citrus pectin. PGC2 was purified from FOC4 through successive steps of ultra filtration, gel filtration chromatography and cation exchange chromatography. During the purification process, the specific PG activity increased from 3.59 to 21.30 units mg protein^-1 ^min^-1 ^(Table 1). One faint single peak of PG activity was seen after culture was applied to a Sephacryl S-100 16/60 gel filtration column. Subjecting the pooled PG fraction to cation exchange chromatography (Sepharose FF CM Hitrap) resulted in a significant single PG peak. SDS-PAGE showed one single protein band and indicated that PGC2 was purified to homogeneity (Figure 1). According to the markers, the molecular weight of PGC2 was about 63 kDa.

**Table 1:** Purification of PGC2 from culture of FOC4 grown on SM supplemented with 1% citrus pectin as the sole carbon source

Step	Total protein (mg)	Total activity (Unit)^a^	Yield (%)	Specific activity (Units mg protein^-1^min^-1^)
Crude	36.60	131.25	100	3.59
Ultrafiltration	8.21	70.26	53.53	8.56
Sephacryl S-100 16/60	1.85	20.35	15.51	11
Sepharose FF CM Hitrap	0.26	5.54	4.22	21.30

^a ^One unit of PG activity was defined as the amount of enzyme that liberated one μmole of GA in one minute at 45°C.

### Characterization of PGC2

The end products of enzymatic hydrolysis of PGA by purified enzyme were analyzed. Galacturonic acid was the only degradation product detected during enzyme activity assays, demonstrating that the purified enzyme is an exoPG.

Treatment of PGC2 with N-glycosidase F reduced the apparent Mr of the enzyme from 63 kDa to 51 kDa, as determined by SDS-PAGE, indicating that PGC2 is a glycoprotein with N-linked carbohydrate (Figure 1). A number of studies have shown that many functional proteins are glycoproteins. Glycosylation has been described in exoPG2 [14] and PG3 [15] from *F. oxysporum *f.sp. *lycopersici*.

**Figure 2:**
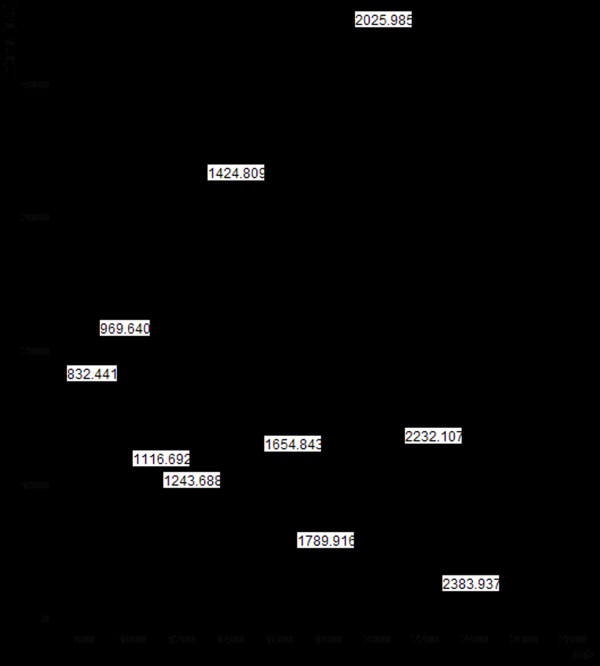
**ESI-MS analysis of the purified PGC2 from FOC4**.

The purified PGC2 was diluted with milli-Q water and filtered through 0.22 μm pore membranes, then subjected to ESI-MS. Mass spectrometry of the purified PGC2 demonstrated many signals (Figure 2) with a molecular mass of 51.7 kDa. The result of ESI-MS also showed that the purified PGC2 may be a glycoprotein, as above.

### Isolation of genes encoding exopolygalacturonase PGC2 from FOC1 and FOC4

The full-length cDNA of *pgc2 *from FOC1 and FOC4 was cloned by 3'-RACE and 5'-RACE. A full-length 1589 nucleotide DNA sequence was isolated and sequenced. Sequencing revealed the presence of an open reading frame (ORF) of 1395 nucleotides, interrupted by four introns of 45, 47, 49 and 49 nucleotides, and encoding a predicted protein of 465 amino acids with a predicted mass of 51.7 kDa and a predicted pI of 8.28. Analysis with SignalP V2.0 detected a putative N-terminal signal peptide sequence of 18 amino acids that would produce a mature protein when cleaved.

The complete nucleotide sequences of *pgc2 *genes from FOC1 and FOC4 were deposited in the GenBank database under accession numbers GU224636 and GU224637.

**Figure 3:**
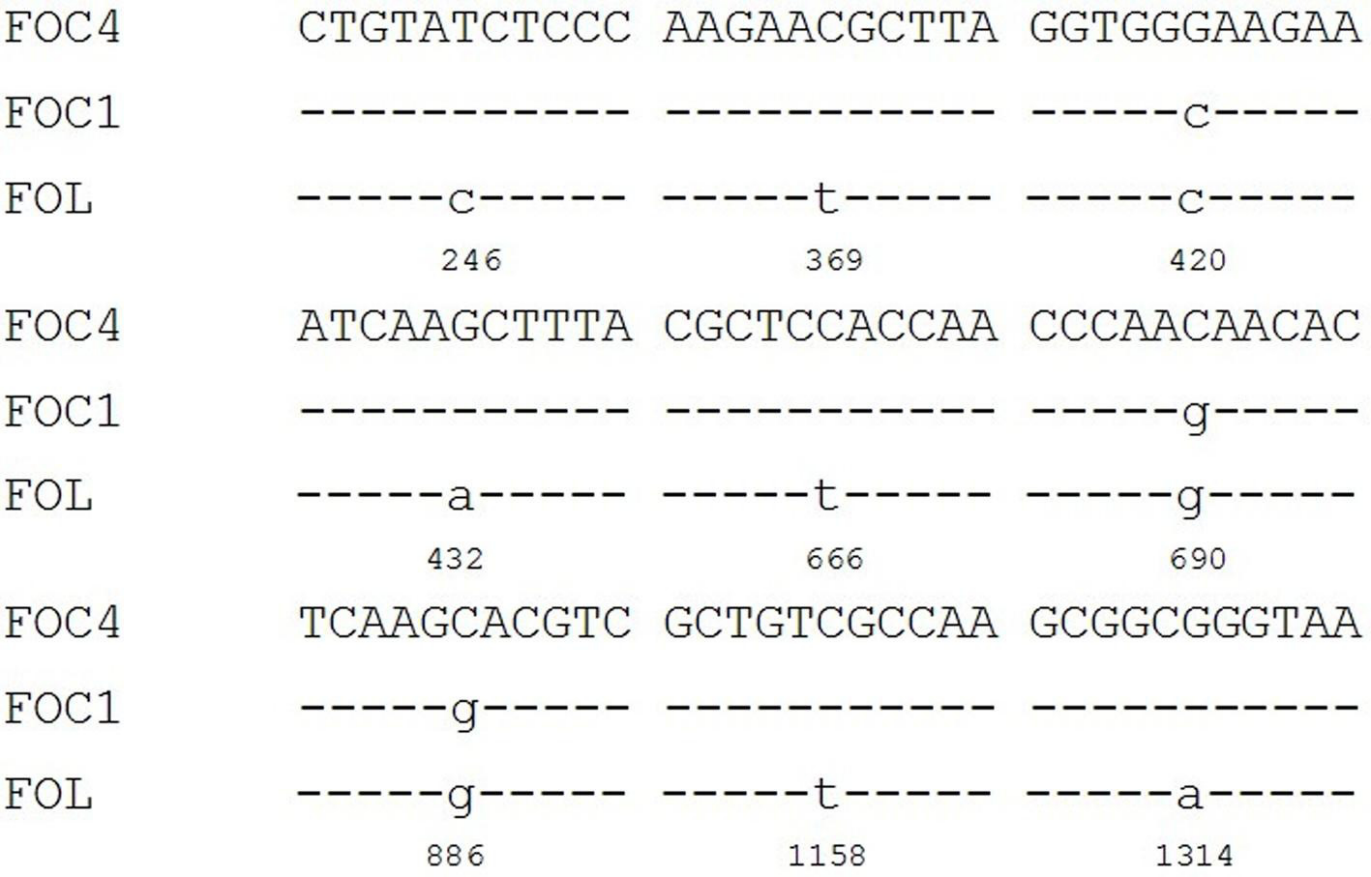
**Multiple nucleotide sequence alignment of *pgc2 *from FOC1 and FOC4 and the *pgx4 *gene from *F. oxysporum *f. sp. *lycopersici *(FOL)**. Sequences analyzed were: FOC4 (GU224637), FOC1 (GU224636), and FOL (AB256795).

The nucleotide sequences of the *pgc2 *genes from FOC1 and FOC4 and the *pgx4 *gene of FOL (accession number: AB256795) [16] were aligned (Figure 3). Among the three nucleotide sequences, nucleotide variation was detected at 9 positions. The sequence of *pgc2 *from FOC4 shared as high as 99.81% and 99.43% nucleotide sequence identity with *pgc2 *from FOC1 and the exoPG gene of FOL, respectively, while the sequence of *pgc2 *from FOC1 shared 99.62% nucleotide sequence identity with FOL. All of the nucleotide variations were located in exons. We noted the following differences between *pgc2*-FOC1 and *pgc2*-FOC4: 420-C to G, 690-G to C and 886-G to C.

**Figure 4:**
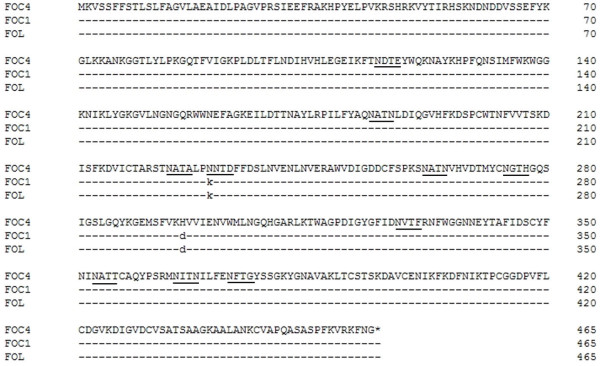
**Multiple amino acid sequence alignment of the predicted proteins of *pgc2 *from FOC1 (**GU224636**), FOC4 (**GU224637**) and exoPG from FOL (**BAE97053**)**. Locations of the *N-*glycosylation site are underlined.

Standard protein-protein BLAST analysis revealed that both isolated PGC2 genes shared a high amino acid sequence identity with exoPGs from other fungi. Figure 4 shows the multiple alignments for the PGC2 amino acid sequences of FOC1, FOC4 and FOL (accession number: **BAE97053**). FOC1-PGC2 shared the same amino acid sequence with FOL and as high as 99.86% amino acid sequence identity with FOC4-PGC2. We noted the following differences between FOC1-PGC2 and FOC4-PGC2: 230-K to N, 296-D to H. Among all prosite motifs, we found a single difference between FOC1-PGC2 and FOC4-PGC2, namely, an *N-*glycosylation site (underlined in Figure 4) present in amino acids 230-233 of FOC4-PGC2 but not in FOC1-PGC2. In other words, there is no *N-*glycosylation site of FOC1-PGC2 at this locus. Whether this variation has an impact on the function of PGC2 is still uncertain.

### Expression and purification of recombinant PGC2

**Figure 5:**
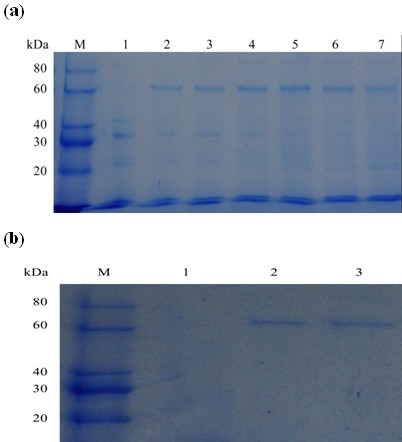
**SDS-PAGE analysis of recombinant PGC2 from FOC1 and FOC4 produced in *P. pastoris***. (a) Cultures were induced for 3 d with methanol as described in Materials and Methods and supernatants from 1, 2, and 3 d were collected. Lane M: protein marker. Lane 1: cultures transformed with pPICZaA. Lane 2-4: culture supernatant from FOC1 at 1, 2, and 3 d. Lane 5-7: culture supernatant from FOC4 at 1, 2, and 3 d. (b) SDS-PAGE analysis of purified PGC2 from FOC1 and FOC4 produced in *P. pastoris*. Lane M: protein marker. Lane 1: cultures transformed with pPICZaA. Lane 2: purified r-FOC1-PGC2. Lane 3: purified r-FOC4-PGC2.

Recombinant PGC2 proteins from FOC1 and FOC4 were expressed in *P. pastoris *as secreted proteins r-FOC1-PGC2 and r-FOC4-PGC2. Culture samples taken at 1, 2 and 3 d post-induction were analyzed by SDS-PAGE (Figure 5a). Proteins of about 63 kDa were detected from the r-FOC1-PGC2 and r-FOC4-PGC2 transformant cultures, but were not observed in control *P. pastoris *transformed with pPICZαA vector.

Specific PG activity was detected from the r-FOC1-PGC2 and r-FOC4-PGC2 transformant cultures but not from control *P. pastoris *transformed with pPICZαA vector. Both recombinant PGC2s were purified through successive steps of (NH_4_)_2_SO_4 _precipitation and gel filtration chromatography. During the purification process, the specific PG activity of r-FOC1-PGC2 and r-FOC4-PGC2 increased from 10.5 to 58.8 units and from 12 to 77.5 units, respectively. SDS-PAGE showed one single protein band for each, indicating that r-FOC1-PGC2 and r-FOC4-PGC2 were purified to homogeneity (Figure 5b). According to the markers, the molecular weight of both recombinant proteins was about 63 kDa.

### Biochemical characterization of recombinant PGC2

**Figure 6:**
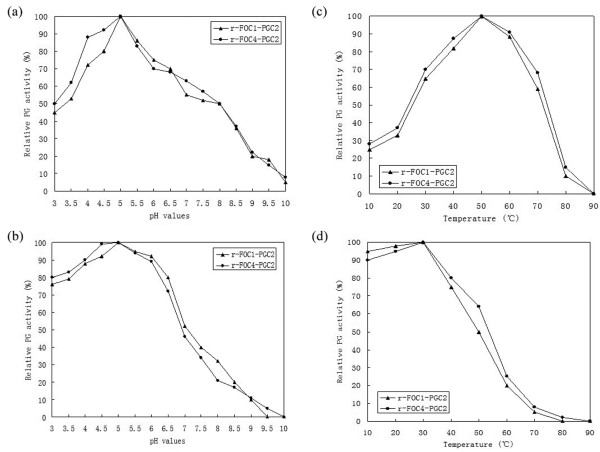
**Enzymatic activity and stability**. (a) Determination of optimal pH. (b) Enzymatic stability with respect to pH. (c) Determination of optimal temperature. (d) Enzymatic stability with respect to temperature. The enzymatic activity and stability were determined as described in the Materials and Methods. One unit of PG activity was defined as one μmol/L of GA released by enzyme min^-1^.

The optimal pH and temperature for PG activity of the recombinant PGC2 proteins were investigated. Both enzymes exhibited the highest activity at pH 5.0 (Figure 6a) and at 50°C (Figure 6b). The pH value and temperature optima for the PG activity from PG2 of FOL were reported to be 5.0 and 55°C [14] and the temperature optimum for the PG activity from PG3 of FOL was 55°C [15].

To estimate the pH stability of recombinant PGC2, samples were incubated in buffers of different pH values at 4°C for 24 h and the remaining PG activity was assayed. Both recombinant PGC2 retained > 70% activity at pH 3-6 (Figure 6c).

To investigate the thermostability of recombinant PGC2, both enzymes were incubated at different temperatures in 100 mM potassium phosphate buffer, pH 5, for 2 h., and then residual activity was determined. Both recombinant PGC2 retained > 50% activity at 10-50°C (Figure 6d).

**Figure 7:**
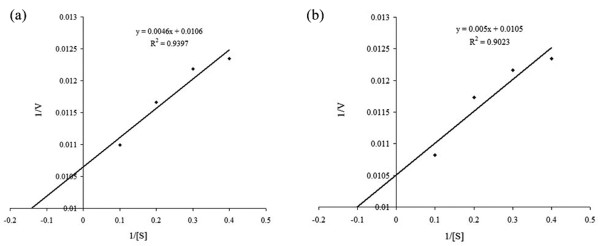
**Lineweaver-Burk plot of purified r-FOC1-PGC2 and r-FOC4-PGC2 activity**. The *K*_m _and *V*_max _values were determined from the Lineweaver-Burk double reciprocal plots of PG activity using PGA as a substrate at concentrations between 2.5 mg ml^-1 ^and 10 mg ml^-1^. (a) Purified r-FOC1-PGC2. (b) Purified r-FOC4-PGC2.

For hydrolysis of polygalacturonic acid (PGA) at pH 5.0 and 45°C, the *K*_m _and *V*_max _of purified r-FOC1-PGC2 were 0.43 mg.mL^-1 ^and 94.34 units mg protein^-1 ^min^-1^, respectively (Figure 7a). The *K*_m _and *V*_max _of purified r-FOC4-PGC2 were 0.48 mg.mL^-1 ^and 95.24 units mg protein^-1 ^min^-1^, respectively (Figure 7b).

### Active recombinant PGC2 causes tissue maceration and necrosis

**Figure 8:**
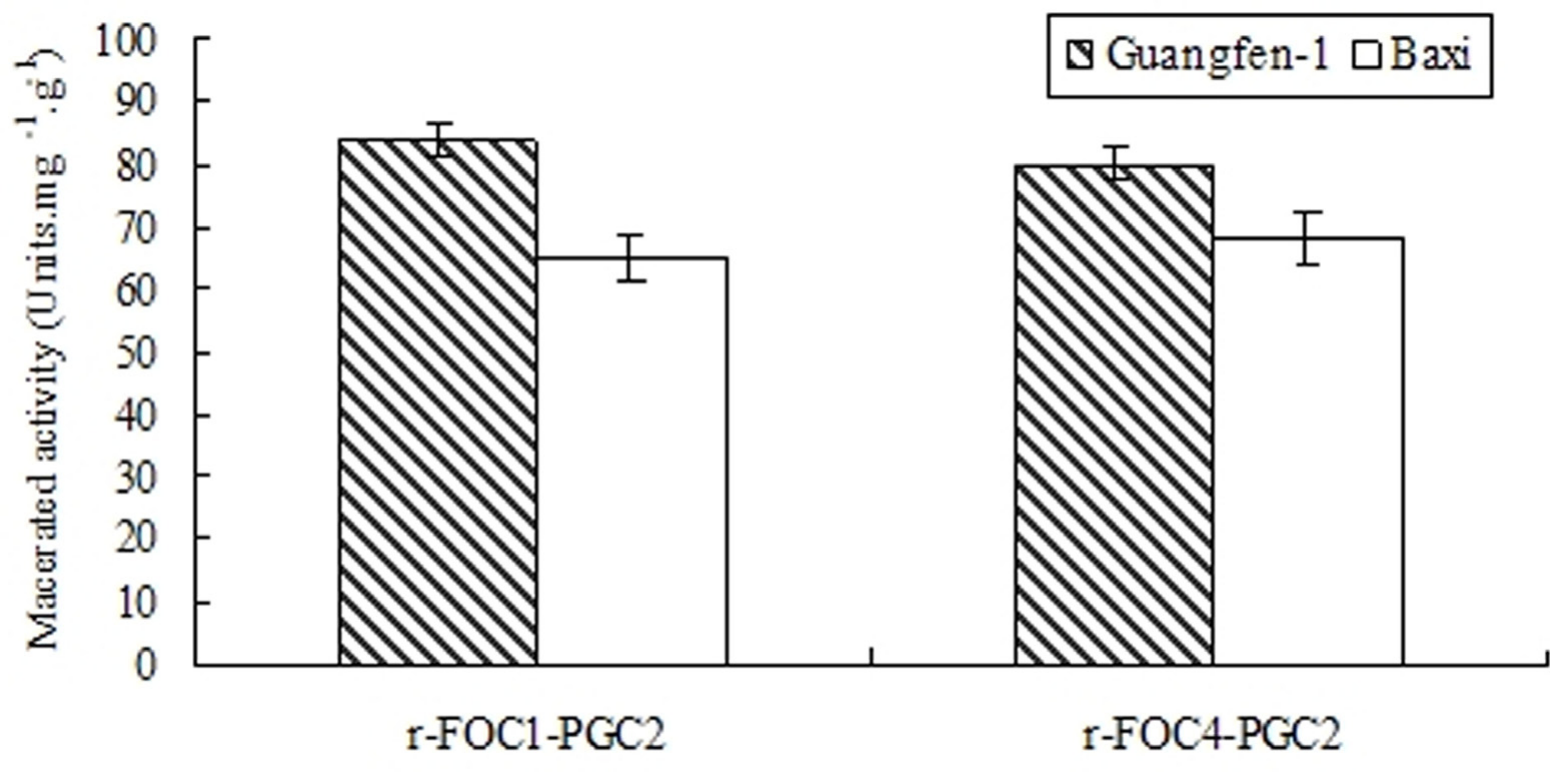
**Enzyme maceration activity in banana tissue**. 1 unit of purified enzyme mixed with 1 ml of 50 mM sodium acetate buffer pH 5.0 was inoculated with sterilized banana tissues, and maceration was evaluated after 48 h at 45°C. Control tubes contained the same buffer without enzyme. The reducing sugar content was determined by the method of Somogyi [18] in three replicates. (Guangfen-1: *Musa *AAB cv. Guangfen-1; Baxi: *Musa *AAA Cavendish cv. Baxi)

Purified r-FOC1-PGC2 and r-FOC4-PGC2 proteins were inoculated onto banana tissues to examine their ability to macerate tissue. Sterilized banana tissues were inoculated with 1 unit of r-FOC1-PGC2 or r-FOC4-PGC2 mixed with 1 ml of 50 mM sodium acetate buffer pH 5.0, and maceration was evaluated after 48 h. The maceration activity of r-FOC1-PGC2 to Guangfen-1 (Musa AAB cv. Guangfen-1) was higher than that of r-FOC4-PGC2, while the maceration activity of r-FOC1-PGC2 to Baxi (Cavendish banana) was lower than that of r-FOC4-PGC2. Both exoPGs showed higher maceration activity on Guangfen-1 than Baxi (Figure 8). These same exoPGs showed differences in their ability to macerate two banana cultivars. These results suggest that pectins of Guangfen-1 and Baxi, as an important component of cell walls, might differ in polymer structure because Baxi pectin is a poor substrate for PGC2 compared to that of Guangfen-1.

**Figure 9:**
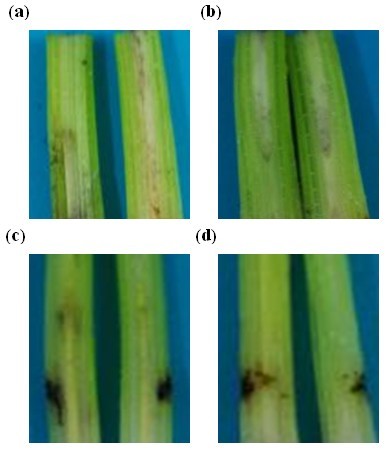
**Tissue necrosis analysis of Baxi banana tissue**. Sterile double distilled water (a) and 50 mM sodium acetate buffer pH 5.0 (b) was used as controls. (c) Banana plants injected with r-FOC4-PGC2. (d) Banana plants injected with r-FOC1-PGC2. Each plant was injected with 1 unit of enzyme. Each treatment was cut (vertical-sectioned) to observe vascular necrosis after 5 d.

Five days after the stems of Cavendish cultivar Baxi plants were injected with 1 unit of r-FOC1-PGC2 or r-FOC4-PGC2, the stem vascular tissues showed partial necrosis (Figure 9). The r-FOC1-PGC2 induced less necrosis compared to r-FOC4-PGC2. It seems that r-FOC1-PGC2 had a lower activity than r-FOC4-PGC2. Sterile double distilled water and 50 mM sodium acetate buffer pH 5.0, used as controls, did not cause any symptoms when injected into stems of the bananas.

## Conclusions

We demonstrated both that the *pgc2 *genes of two races of FOC could be expressed in *P. pastoris *and that the recombinant proteins displayed PG activity. The two exoPGs have similar molecular structures and almost the same enzyme characteristics. Further studies of these gene products will lead to valuable insights into the role of PGC2 in FOC pathogenicity in banana cultivars.

## Methods

### Fungal strains and growth conditions

*F. oxysporum *f. sp. *cubense *race 4 (FOC4) was obtained from Guangzhou, China, and *F. oxysporum *f. sp. *cubense *race 1 (FOC1) was obtained from Nanning, China. The pathotype of the isolates was periodically confirmed by plant assays in a growth chamber. The cultivars tested comprised *Musa *AAA Cavendish cv. Baxi, resistant to FOC1 and susceptible to FOC4; *Musa *AAB cv. Guangfen-1, susceptible to FOC1 and FOC4.

For pectinolytic enzyme production and extraction of RNA, fungal strains were grown in SM [17] supplemented with 1% [w/v] citrus pectin (Sigma). For extraction of DNA, mycelium was obtained from cultures grown for 5 d in potato dextrose broth (PDB) in Erlenmeyer flasks on a rotary shaker at 110 rpm and 25°C.

### Purification of PGC2

Cultures of FOC4 grown in SM supplemented with 1% citrus pectin were centrifuged at 16,000 *g *for 20 min. Supernatant was collected and concentrated using an Amicon 8400 ultrafiltration system containing a 10 kDa MWCO membrane.

Concentrated extract filtrate was applied to a gel filtration column (Sephacryl S-100 16/60, Pharmacia) equilibrated and eluted with 50 mM sodium acetate buffer (pH 4.5) at a flow rate of 1 ml/min. Fractions containing PG activity were pooled and applied to a cation exchange column (Sepharose FF CM Hitrap, Pharmacia) equilibrated with 20 mM sodium acetate buffer (pH 4.5). The column was eluted with a gradient of NaCl (0-0.7 M) at a flow rate 2 ml/min.

### PG activity and protein assays

PG activity was routinely determined by measuring the release of reducing groups from polygalacturonate (PGA; Sigma). The standard reaction mixture (1 ml total volume) contained 0.5% PGA (w/v), 50 mM sodium acetate buffer (pH 5.0), and various amounts of the enzyme preparation. After incubation at 45°C for 30 min, the reducing sugar content was determined by the methods previously reported [18]. Appropriate controls without either enzyme or substrate were run simultaneously. The quantity of reducing sugar released was calculated from standards of D-galacturonic acid (GA; Sigma). One unit of enzyme activity was defined as the amount of enzyme that released 1 μmol of GA equivalent per minute under the above conditions.

Discontinuous sodium dodecyl sulfate-polyacrylamide gel electrophoresis (SDS-PAGE) was performed according to Laemmli [19] using 10% acrylamide. After electrophoresis, gels were stained with Coomassie brilliant blue G250 (Sigma). Protein concentration was determined according to the Bradford method [20] using bovine serum albumin as a standard.

### Biophysical characterization of the PGC2

The purified PGC2 was resolved on SDS-PAGE, blotted onto a PVDF membrane and analyzed by electrospray ionization-mass spectrometry (ESI-MS) using a triple quadrupole instrument from Applied Biosystems, API 3000 (PE Sciex, Canada). Samples were diluted in milli-Q water with a saturated solution of a-cyano-4-hydroxycinnamic acid containing 50% (v/v) acetonitrile and 0.1% (v/v) TFA. The spectra were acquired over a mass/charge (m/z) range of 400-2000 in direct and reflective mode and were interpreted using Voyager software v.5.0.

### Biochemical characterization of PGC2

For analysis of hydrolysis products, the purified enzyme (0.02 units in 0.5 ml distilled water) was added to 1 ml of 0.5% (w/v) PGA in 50 mM sodium acetate buffer (pH 5), incubated at 45°C for 10, 20, 30, 40, 50, or 60 min, and then assayed for PG activity. Removal of *N*-linked carbohydrate was carried out using N-glycosidase F (Boehringer Mannheim, Mannheim, Germany). Deglycosylation protocols followed the manufacturer's instructions.

The standard assay was modified to test the effects of varying pH and incubation temperature. To determine the optimal pH, enzyme activity was assayed using 100 mM potassium phosphate buffer for pH values between 3 and 10 at 45°C and 0.5% (w/v) PGA as substrate. The effect of temperature on PGC2 activity was determined in the same buffer at pH 5 between 10°C and 90°C. One unit of PG activity was defined as one μmol/L of GA released by enzyme min^-1^.

To estimate pH stability, samples were incubated in 100 mM potassium phosphate buffer of different pH values at 4°C for 24 h. To evaluate the thermal stability, protein samples were incubated at different temperatures in the same buffer at pH 5 for 2 h. The residual activity was determined as previously described.

The *K*_m _and *V*_max _values were determined from the Lineweaver-Burk double reciprocal plot of PG activity using PGA as substrates at concentrations between 2.5 mg ml^-1 ^and 10 mg ml^-1^. The activity was assayed as previously described.

### Cloning of fungal *pgc2 *genes

For RACE PCR, total RNA from FOC4 was reverse-transcribed into cDNA with murine leukemia-virus reverse transcriptase (TaKaRa). Two oligonucleotide primers were designed based on the *N*-terminal partial sequence of PGC2. The first PCR used the degenerate sense primer P1: 5'-GCNATHGAYYTNCCNGC-3', nest-PCR primer nP1: 5'-CCNGCNGGNGTNCCNMG-3', with the antisense primer Oligo(dT)20. Amplification cycles were as follows: one cycle of 3 min at 94°C, 35 cycles of 94°C for 30 s, 48°C for 30 s, and 72°C for 90 s, then 10 min for 72°C. The amplified fragments were gel-purified and cloned into pMD18-T (TaKaRa) and sequenced. Two primers (P2: 5'- GTAGTAGCGTTGATGTTG-3', nP2: 5'-GTAGCACGAGTCGATG-3') was designed for 5'-RACE in accordance with RT-PCR sequence, which was performed essentially as described for the 5' -Full RACE Core Set (TaKaRa).

Based on the nucleotide sequence of the RACE PCR, first-strand cDNAs were synthesized from total RNA of FOC1 and FOC4 using reverse transcriptase XL (TaKaRa) by reverse transcription polymerase chain reaction (RT-PCR) using the forward primer (5'-GTGGAATTCATGAAGGTCTCGAGCTTCTTCT-3') and the reverse primer (5'- CGGTCTAGATTAACCGTTGAACTTTCTAACC-3'). The *Eco*RI and *Xba*I restriction sites are underlined. The following PCR conditions were used: 35 cycles with denaturation at 94°C for 30 s, annealing at 49°C for 30 s and extension at 72°C for 90 s. An initial denaturation step of 4 min at 94°C and a final elongation step at 72°C for 10 min were performed. The RT-PCR products were cloned into pMD18-T (TaKaRa) and verified by nucleotide sequencing analysis, and then they were digested with *Eco*RI and *Xba*I and subcloned into the recombinant eukaryotic expression vector pPICZaA (Invitrogen) digested with the same enzymes to generate pPICZaA-*pgc2*-FOC1 and pPICZaA-*pgc2*-FOC4. The presence of the inserted *pgc2*-FOC1 and *pgc2*-FOC4 genes was verified by nucleotide sequencing analysis.

Genomic DNA was extracted from fungal mycelium using the E.Z.N.A Fungal DNA Kit (OMEGA) according to the manufacturer's instructions. DNA sequences were sequenced using DNA isolated from FOC1 and FOC4 as the template and primers described above.

The complete nucleotide sequences of *pgc2 *genes from FOC1 and FOC4 were deposited in the GenBank database under accession numbers **GU224636** and **GU224637**.

### Expression and purification of PGC2 in *P. pastoris*

Yeast transformation was performed according to manufacturer's instructions. The recombinant plasmids pPICZaA-*pgc2*-FOC1 and pPICZaA-*pgc2*-FOC4 were linearized by digestion with *Sac*I and transformed into *P. pastoris *SMD1168 strain by electroporation. SMD1168 strains transformed with pPICZaA plasmid and SMD1168 strains without transformation served as negative controls. Cells were incubated on yeast extract peptone dextrose (YPD) plates containing 1% yeast extract, 2% peptone, 2% dextrose, and 100 μg/ml of zeocin at 28°C for 48 h. Integration of the *pgc2 *gene into the genome of *P. pastoris *was determined by PCR using 5'AOX1 and 3'AOX1 primers. The presence of the *pgc2 *gene in transformants was confirmed by gene sequencing of PCR products.

Yeast transformants were grown in 30 ml of BMGY medium (1% yeast extract, 2% peptone, 1.34% yeast nitrogen base, 100 mM potassium phosphate, 4 × 10^-5^% biotin and 1% glycerol) at 28°C for 24 h. Cell pellets were harvested and resuspended in 200 ml BMMY (1% yeast extract, 2% peptone, 1.34% yeast nitrogen base, 100 mM potassium phosphate, 4 × 10^-5^% biotin and 0.5% methanol). The cultures were returned to the incubator and grown under the same conditions, and samples were collected at 1, 2 and 3 d. 1 ml of 100% methanol was added every 24 h to maintain a final concentration of 0.5%, assuming that the methanol was completely utilized in 24 h.

The cultures were centrifuged at 10,000*g *for 20 min at 4°C, and supernatants were filtered through membranes (0.22 μm, Millipore). The supernatant was fractionated by salting out with solid ammonium sulfate at 0-80% (w/v) saturation. The precipitate formed was collected by centrifugation at 10,000*g *for 20 min at 4°C, dissolved in 50 mM sodium acetate buffer (pH 5.0), and then dialyzed overnight against the same buffer at 4°C.

After centrifugation, recombinant r-FOC1-PGC2 and r-FOC4-PGC2 were further purified using a gel filtration column (Sephacryl S-100 16/60, Pharmacia) equilibrated and eluted with 50 mM sodium acetate buffer (pH 5.0) at a flow rate of 1 ml/min. Fractions containing PG activity were collected.

### Tissue maceration and necrosis assayed with recombinant PGC2

To evaluate tissue maceration, the cultivars tested comprised *Musa *AAA Cavendish cv. Baxi, resistant to FOC1 and susceptible to FOC4; *Musa *AAB cv. Guangfen-1, susceptible to FOC1 and FOC4. 1 cm lengths of tissue (0.5 g) were taken from the healthy stems of the four leaf stage banana and placed in test tubes. A mixture of 1 unit of purified enzyme with 1 ml of 50 mM sodium acetate buffer, pH 5.0, was inoculated with the sterilized banana tissue, and maceration was evaluated after 48 h. Control tubes contained the same buffer without enzyme. Released reducing sugar was calculated from standards of GA after incubation 48 h at 45°C. The reducing sugar content was determined by the method of Somogyi [18].

For the tissue necrosis assay, 1 unit of enzyme was applied to the stems of healthy banana plant by injection. For each treatment, stems were cut (vertical-sectioned) 5 d later to observe vascular necrosis, ten replicates. Sterile double distilled water and 50 mM sodium acetate buffer pH 5.0 were used as controls.

## Abbreviations

FOC4: *Fusarium oxysporum *f.sp. *cubense *race 4; FOC1: *Fusarium oxysporum *f.sp. *cubense *race 1; FOL: *Fusarium oxysporum *f.sp. *lycopersici*; PG: polygalacturonase; ExoPG: exopolygalacturonase; PGC2: An exopolygalacturonase of *Fusarium oxysporum *f.sp. *cubense *race 4; PGA: polygalacturonic acid.

## Authors' contributions

ZW defined the concept and experiments of this study. ZD performed research and analyzed data. ZD and ZW drafted the manuscript. ZD and ZW have read and approved the final manuscript.

## References

[B1] Heslop-Harrison JS Schwarzacher T Domestication, genomics and the future for banana Ann Bot-London 2007 100 1073 1084 10.1093/aob/mcm191 PMC2759213 17766312

[B2] Cheesman E Pathology of the Banana Nature 1962 194 223

[B3] Ploetz RC Fusarium wilt of banana is caused by several pathogens referred to as Fusarium oxysporum f. sp cubense Phytopathology 2006 96 653 656 10.1094/PHYTO-96-0653 18943184

[B4] Persley GJ Banana and Plantain Breeding Strategies. Proceedings of an International Workshop Held at Cairns 1987 Australia. State Mutual Book & Periodical Service, Ltd

[B5] Hwang SC Ko WH Cavendish banana cultivars resistant to fusarium wilt acquired through somaclonal variation in Taiwan Plant Dis 2004 88 580 588 10.1094/PDIS.2004.88.6.580 30812575

[B6] Grimm D PLANT GENOMICS A Bunch of Trouble Science 2008 322 1046 1047 10.1126/science.322.5904.1046 19008426

[B7] Walton JD Deconstructing the cell-wall Plant Physiol 1994 104 1113 1118 12232152 10.1104/pp.104.4.1113 PMC159271

[B8] RB WR Cooper RM Cell wall-degrading enzymes of vascular wilt fungi. II. Properties and modes of action of polysaccharidases of Verticillium albo-atrum and Fusarium oxysporum f. sp. lycopersici Physiol Mol Plant P 1978 13 101 134 10.1016/0048-4059(78)90079-6

[B9] Riou C Freyssinet G Fevre M Purification and Characterization of Extracellular Pectinolytic Enzymes Produced by Sclerotinia sclerotiorum Appl Environ Microbiol 1992 58 578 583 16348646 10.1128/aem.58.2.578-583.1992 PMC195287

[B10] Scott-Craig JS Cheng YQ Cervone F De Lorenzo G Pitkin JW Walton JD Targeted mutants of Cochliobolus carbonum lacking the two major extracellular polygalacturonases Appl Environ Microbiol 1998 64 1497 1503 9546185 10.1128/aem.64.4.1497-1503.1998 PMC106176

[B11] Garcia-Maceira FI Di Pietro A Roncero MI Cloning and disruption of pgx4 encoding an in planta expressed exopolygalacturonase from Fusarium oxysporum Mol Plant Microbe 2000 13 359 365 10.1094/MPMI.2000.13.4.359 10755298

[B12] Favaron FAPM Polygalacturonase isozymes and oxalic acid produced by Sclerotinia sclerotiorum in soybean hypocotyls as elicitors of glyceollin Physiol Mol Plant P 1988 385 395

[B13] de Cervone FLGP Can Phaseolus PGIP inhibit pectic enzymes from microbes and plants? Phytochemistry 1990 447 449

[B14] Di Pietro A Roncero M Purification and characterization of an exo-polygalacturonase from the tomato vascular wilt pathogen Fusarium oxysporum f. sp. lycopersici Fems Microbiol Lett 1996 145 295 299 10.1111/j.1574-6968.1996.tb08592.x 8961570

[B15] Garcia Maceira FI Di Pietro A Roncero MIG Purification and characterization of a novel exopolygalacturonase from Fusarium oxysporum f.sp. lycopersici Fems Microbiol Lett 1997 154 37 43 10.1016/S0378-1097(97)00298-X

[B16] Hirano Y Arie T PCR-based differentiation of Fusarium oxysporum f. sp. lycopersici and radicis-lycopersici and races of F-oxysporum f. sp. lycopersici Journal of General Plant Pathology 2006 72 273 283 10.1007/s10327-006-0287-7

[B17] DiPietro A Roncero M Endopolygalacturonase from Fusarium oxysporum f. sp. lycopersici: Purification, characterization, and production during infection of tomato plants Phytopathology 1996 86 1324 1330

[B18] Smogyi M Notes on sugar determination J Biol Chem 1952 195 14938350

[B19] Laemmli UK Cleavage of structural proteins during the assembly of the head of bacteriophage T4 Nature 1970 227 10.1038/227680a0 5432063

[B20] Bradford MM Rapid and sensitive method for quantitation of microgram quantities of protein utilizing principle of protein-dye binding Anal Biochem 1976 72 248 254 10.1016/0003-2697(76)90527-3 942051

